# Bird diversity along riverine areas in the Bhagirathi Valley, Uttarakhand, India

**DOI:** 10.3897/BDJ.7.e31588

**Published:** 2019-04-30

**Authors:** Ankita Sinha, Hima Hariharan, Bhupendra Singh Adhikari, Ramesh Krishnamurthy

**Affiliations:** 1 Wildlife Institute of India, Dehradun, India Wildlife Institute of India Dehradun India; 2 Salim Ali Center for Ornithology and Natural History, Coimbatore, India Salim Ali Center for Ornithology and Natural History Coimbatore India; 3 Wildlife Institute of India, Dehardun, India Wildlife Institute of India Dehardun India

**Keywords:** Western Himalaya; riverine forests; Bhagirathi; habitat; elevational gradient, avifauna

## Abstract

Natural riverine areas mark ecotonal habitats harbouring a characteristically diverse faunal assemblage, especially birds that also use these habitats as pathways crucial for their movement. Increasingly, riverine systems are subjected to large-scale habitat alterations due to climatic fluctuations and anthropogenic changes. Therefore, it is important to understand broad-scale community patterns for conservation planning and prioritisation for these ecotone habitats. The Bhagirathi river is one of the major headwaters of the river Ganges; despite its rich and diverse fauna, little is known about the bird species that inhabit this montane region. This study presents an extensive list of 281 bird species from 59 families, their seasonal distribution and habitat associations as recorded from field surveys along the riverine areas between April 2013 and May 2018. The present communication simultaneously discusses a few noteworthy sightings for the region and provides a baseline for future research on the distribution of birds in the Western Himalaya.

## Introduction

Natural riverine areas encompass interfaces between land-aquatic systems with sharp environmental gradients representing the most diverse, dynamic and complex biophysical habitats on earth (Naiman et al. 1993). Although riparian corridors are well known for their high levels of biodiversity, the values have seldom been quantified. While riparian zones typically are a small component of the landscape, they provide an essential habitat for many species of birds (Knopf 1985, Stauffer and Best 1980, Stevens et al. 1977). Riverine systems, being prone to large-scale habitat alterations due to natural and climatic fluctuations, call for devising potential indicators for monitoring ecosystem health. Birds are conspicuous, operate at multiple scales and often occupy apex positions in food webs. Thus, they suffice as potential candidates for long-term monitoring purposes, especially through popular citizen-science programmes. Riparian ecotones often support an avian community that is more diverse and with a higher abundance than the surrounding uplands (Gates and Giffen 1991, Stauffer and Best 1980). Riverine forests also support high densities and diversities of migratory birds providing pathways and edge cover during migration (Gergel et al. 2002, Naiman et al. 1993). In addition, species may use riparian areas differentially throughout the season (Rice and Anderson 1980); hence, habitat associations of different species need to be monitored across seasons to thoroughly appraise riparian zones for conservation.

The Himalayan mountain system is globally renowned for its notable biological diversity, supported by the complex orogeny and consequent climatic and edaphic conditions. The avifauna of the Western Himalaya, an Endemic Bird Area (Jathar and Rahmani 2006), has attracted a number of ornithologists and naturalists over the years. Birds inhabiting this region show a large variety of distributional patterns with some species being restricted to narrow elevational bands while others are relatively broadly distributed. Amongst these, a large number undertake short migration from higher elevation breeding grounds to warmer lower elevations for wintering (Grimmett et al. 2013). Thus, the avifaunal assemblage of any particular location remains dynamic.

Habitat alteration remains a major threat to montane ecosystems around the world, the phenomenon being pronounced in the Himalaya. Parallel to being biodiverse, freshwater systems are abode to millions of human population, the Ganges being the most densely populated river basin of the world (Immerzeel et al. 2010). Distortion of land and water due to developmental projects and increasing agricultural pressure is well documented in this region (Grumbine and Pandit 2013, Manel et al. 2000) and warrant a dire need to document the floral and faunal diversity of natural versus modified landscapes. Misapprehending the risks involved in land-use decisions, including the construction of hydroelectric dams in the Indian Himalaya (Bandyopadhyay 1995), can lead to large scale negligence towards biodiversity conservation strategies. Biodiversity loss has multiple causes, but habitat destruction via land-use change has remained a predominant driver (Butchart et al. 2010, Sala et al. 2000).

In this study, we inventoried the avifauna of the Bhagirathi Valley in the Western Himalayan Region, India. There exists no previous published literature concerning avifauna for this region. We documented bird species occurring in the region during pre- and post-monsoon seasons along with reporting of some opportunistic records. We have discussed the habitat associations of the recorded bird species along with their seasonal distribution in the river basin. We also report some noteworthy sightings which are new to this region and the state. This is the first published multi-year study of distribution patterns of birds from the Bhagirathi valley, Uttarakhand, India.

## Material and methods

**Figure 1.**
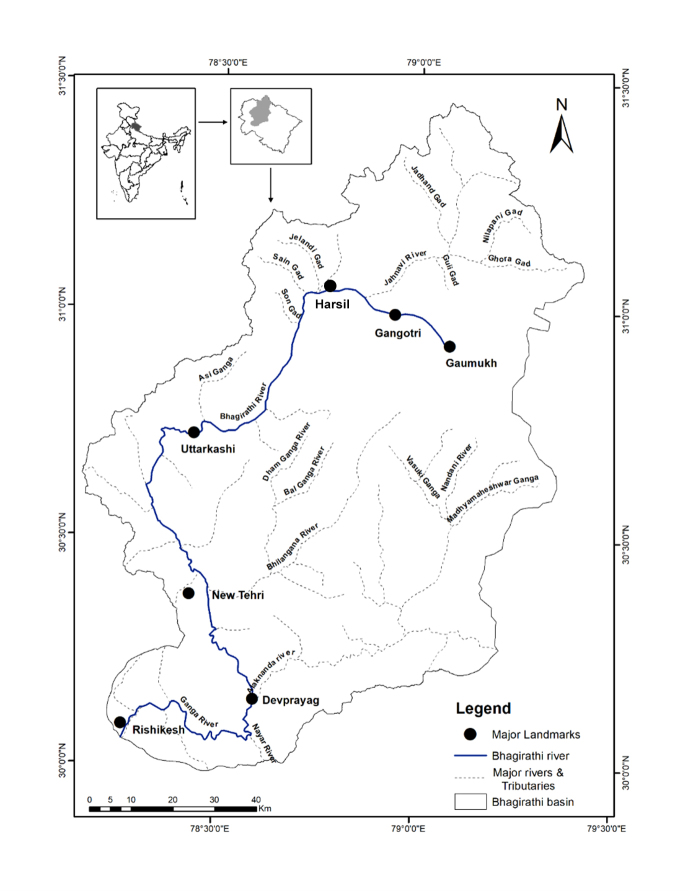
Map of the Bhagirathi basin, in the state of Uttarakhand, India showing the Bhagirathi river, important tributaries and major towns along the river

The Himalaya encompasses the highest mountains in the world; snow and glacier melt run-off being the major source of water for the Himalayan rivers. Biogeographically, this enormous mountain range has been divided into Northwestern-, Western-, Central-, Eastern- and Trans-Himalayan regions (Rodgers and Panwar 1988). This study was conducted along the river Bhagirathi, one of the major headstreams of the Upper Ganges in the state of Uttarakhand in the Western Indian Himalaya. Field surveys were undertaken along a 217 km river stretch, between an elevational gradient of 330 m asl (30.11775°N, 78.30722°E) in Rishikesh and 3,200 m asl (30.99419°N, 78.94388°E) in Gangotri (Fig. 1).

The catchment has mean summer temperatures of 1.3°C–39.6°C and winter temperatures of -27.4 to 7.6°C, while annual precipitation ranges from 533–2,284 mm. Given the large altitudinal relief, the study area is characterised by diverse biomes. The river flows through deep gorges and narrow valleys and is lined by different land-uses ranging from agriculture to urban sprawls. The development of Tehri dam, Koteshwar hydropower plant and Kotli-Bhel hydropower project (under development in Bhagirathi basin) has led to the diversion of approximately 68 km (31%) of the river Bhagirathi; around 85 km (39%) of the riverine buffer zone has been submerged to a width of 1 km (Rajvanshi et al. 2012). Along forested areas, the major tree species in riverine areas include conifers in the higher elevations (above 2300 m asl), like *Cedrus deodara*, *Picea smithiana*, *Pinus wallichiana* and *Pinus roxburghii* and other broadleaf riverine specialists like *Populus ciliata* around Harsil and Gangotri. Other significant riverine trees in the middle (1200-2000 m asl) elevation include *Alnus nepalensis* and *Toona ciliata* around Uttarkashi. Around backwaters of the Tehri dam, plantations of *Pinus roxburghii* dominate, along with patches of *Acacia catechu* and *Dalbergia sisoo*. Mixed forests dominate riverine stretches along lower (300-700 m asl) elevations; around Rishikesh and Devprayag, dominant species being *Bauhinia variegata*, *Mallotus philippinensis*, *Haldina cordifolia*, *Shorea robusta* and *Holoptelea integrefolia*.

A pilot survey was conducted in the study area to understand the different habitat types present and the utilisation of those habitat types by various bird species. For every sighting, the habitat use by individual birds was noted and behaviour was classified as feeding, roosting or nesting. Bird checklists were meticulously maintained in all the accessible areas along the river around Rishikesh (300 m asl), Devprayag (700 m asl), New Tehri (2,100 m asl), Uttarkashi (1,300 m asl), Harsil (2,500 m asl) and Gangotri (3,200 m asl). Exhaustive bird lists were made during pre-monsoon (February-June) and post-monsoon (September-January) seasons at each of these locations between April 2013 and May 2018. A total of 72 trails of 500 m length each were walked at different times of the year by a single observer every time. Out of these, 41 were permanent which were sampled thrice every season for all the years. Apart from these, opportunistic sightings were also noted. Both vocalisations and direct sightings were used for bird identification. Photographs were taken on all possible occasions for future reference and especially for rare species previously unrecorded from this region. The identification of birds was based on standard literature (Grimmett et al. 2013) and the names were listed following (Praveen et al. 2018). To understand broad patterns of habitat use by different bird species, birds were classified into three major functional categories; (A) riverine: habitat especially or generally near water, (B) riparian: riparian or water mentioned in habitat accounts and (C) terrestrial: woodlands, grasslands or no mention of water in habitat accounts, based on field observations and literature collated from Ali and Ripley (1968).

## Results

**Figure 2.**
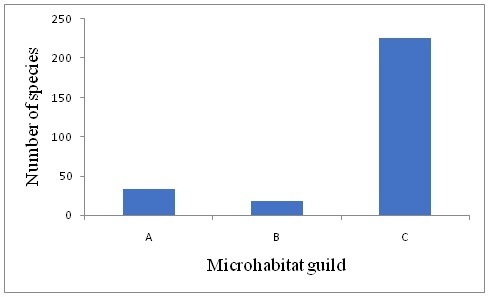
Microhabitat guilds of the birds recorded during the survey period in the Bhagirathi basin; A: Especially or generally near water; B: Riparian or water mentioned in habitat counts; C: Woodlands, grasslands, no mention of water in habitat accounts.

**Figure 3.**
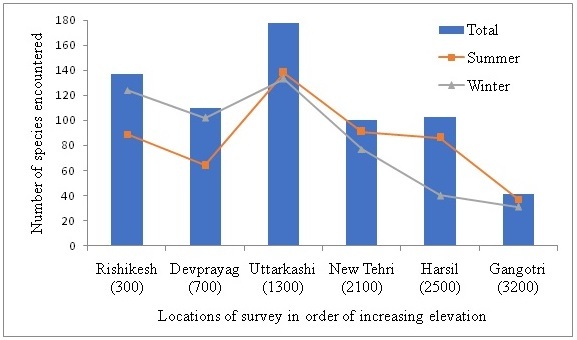
Total bird species richness and turnover across seasons at different elevations (m asl) sampled along the river Bhagirathi. Mid-elevations around Uttarkashi showed maximum species richness and showed least species turnover. Lower elevations around Rishikesh and Devprayag (300-700 m asl) showed moderate species richness and high species turnover. Very high elevation around Gangotri showed less species richness.

A diverse population of birds belonging to 64 families were identified in the riverine areas along the Bhagirathi river (upper Ganges) at different elevations during the survey period. A total of 280 bird species were encountered during the survey period which constitutes almost 40% of the total number of species (693) reported from the state of Uttarakhand (Mohan and Sondhi 2015). Muscicapidae (30 species) followed by Accipitridae (18 species) were the most dominant families in the study area. Other families with significant representation were Fringillidae (13 species), Picidae (13 species), Corvidae (11 species) and Turdidae (10 species). Species from upland forests add to the species diversity in the riparian corridor (Fig. 2), especially insectivores reflected in the large representation from the Muscicapa, Turdidae and Leothricidae families (Suppl. material 1Table 1). Over 30 bird species, that are solely dependent on the river or use it opportunistically, were recorded during this survey period (Fig. 2). The maximum number of species recorded were terrestrial, with no mention of water in habitat accounts (Fig. 2). Bird species richness varied greatly with elevation and across seasons. Maximum number of species (n=178) were recorded in mid-elevation sites in and around Uttarkashi and the least (n=41) were recorded in high elevation sites around Gangotri (Fig. 3). Species richness was consistently higher in the lowest elevations (Fig. 3), although some species, residing around the elevations between 300 to 700 m asl, were not hill birds, such as the Jungle Babbler, Indian Peafowl, Red-vented Bulbul, Spotted Dove and Brown-headed Barbet (Table 1). Although few species were recorded at high elevations in winter, species richness was high around Harsil (at 2,500 m asl) in summer. In winter, species richness decreased sharply with elevation (Fig. 3).

There was a large amount of seasonal turnover at each location, demonstrating substantial elevational migration undertaken by a large fraction of the avian community (Fig. 3). Lower elevations (Rishikesh and Devprayag) showed more species in winter, where a number of species used/preferred these habitats as wintering grounds to escape the harsh winters. This pattern is common and has been observed in previous studies in Western Himalayas with mid- and high-elevation habitats experiencing high species turnover between winters and summers (Somveille et al. 2013).

Ornithologically noteworthy sightings from the region are discussed below.

Ibisbill (Fig. 4d): During our study period, Ibisbill was recorded at multiple incidents around Harsil and Dharali at an elevation of 2,500 m asl. Adults, sub-adults and chicks were seen on multiple occasions. Adults were recorded year-round feeding from shingle beds near Bagori village and Dharali in the Harsil valley. Breeding grounds of Ibisbill were documented in Harsil and our records qualify this population as resident (Sinha et al. 2015). Insights on the ecology and population status of this elusive riverine obligate bird species holds potential for study.

Cheer Pheasant (Fig. 4c): Apart from nine other members of the Phasianidae family (Table 1,Suppl. material 1), the endangered Cheer Pheasant (Endangered, IUCN Red List) was recorded at two sites. On one occasion, a pair (male and female) was seen during low light period of early morning hours (near Gangani, Uttarkashi district) at an elevation of 2,200 m asl on 11 November 2017. The birds were spotted on the highway basking in the sun, the habitat being dominated by tall grass, dense bushes and oak (*Quercus leucotrichophora*) - rhododendron (*Rhododendron arboreum*) forests. The birds were very shy and flew off immediately. On another occasion, a single individual was photo-captured in a pine forest near Devprayag (850 m asl). The bird, being a habitat specialist, requires open, early successional habitats in the Himalaya. The bird remains in few small refuges with its habitat being heavily disturbed (Garson et al. 1992).

Demoiselle Crane (Fig. 4a): A single bird was seen in Harsil in late May 2014 at around 5:00 p.m. The bird was feeding voraciously by pecking on insects from the river bed while walking for small stretches intermittently. It continued feeding till light faded. It was seen in this locality for two consecutive days though there were no further sightings. This individual might be a vagrant which used this site as a stopover during the long migration back to the Mongolian highlands. The species is a new record at this altitude (2,500 m asl) for the state of Uttarakhand.

Northern Shoveller: A single bird was seen in early March 2018 in Dharali (near Harsil at 2,600 m asl), often roosting along vegetated banks of the main river channel in a pool-like stretch where flow was not fast. Groups of two to three birds were seen in the backwaters of the Maneri dam in winters of 2013, 2014 and 2017.

Northern Goshawk (Fig. 5b): A single bird was seen on 9 November 2017 in Harsil chasing a Green-backed Tit in broad daylight hours around 10:30 a.m. along a small stream. It manoeuvred efficiently, confirming its tactics of surprise hunting by flying swiftly amidst houses, shrubs and tall trees. The species is known to prefer well vegetated broadleaf and coniferous forests at high elevations almost up to treeline in the Himalaya (Ali and Ripley 1968). Habitat accounts often mention vicinity to stream and riverine areas.

Golden Bush Robin (Fig. 5f): A single bird was seen in winter and a pair (male and female) in spring in thickets, with dense undergrowth and scattered *Rhododendron arboreum* in Uttarkashi (1,300 m asl). They breed in alpine Rhododenron shrubs and winter to lower elevations.

Desert Wheatear (Fig. 5a): A pair of females was spotted on sandy river beds in Harsil on 12 April 2018. This eastern sub-species is known to breed in large parts of central Asia and winters further south. This record is unusual as both the individuals were seen in breeding plumage and there are no previous breeding or wintering records of this species from this area.

Wallcreeper (Fig. 5e): Seen at multiple locations around Rishikesh, Devprayag and Uttarkashi on river beds in winter. Birds fed at riverine stretches with gorges, vertical cliffs, especially near streams or small cascades, earthen walls, concrete walls, buildings and archaeological ruins and boulders in river beds.

Large-billed Leaf Warbler: The species was recorded breeding in summer in Harsil. It occupied coniferous forests, almost invariably in the vicinity of torrential streams. They were usually seen foraging from top canopy, but often in the middle canopy of very tall deodars. Birds were frequently sighted singly or in pairs along the stream under overhanging bank with tangled roots of fallen trees often overlooking a stream.

Black-throated Sunbird: A single male bird was seen in Uttarkashi, Maneri, around an elevation of 1,300 m asl amidst human settlement with plantations. Bird was seen voraciously feeding from blooms of *Callistemon* (bottle-brush) with frequent trills on an overcast day (21 March 2018). Reported sporadically from Uttarakhand, this is probably the western-most distribution record for this species.

Red-headed Bullfinch (Fig. 4b): A flock of six birds were seen on multiple days in February 2014 in Dharali (Harsil) at an elevation of 2,600 m asl. The birds were feeding from dry branches on a snowy day. Another sighting was in spring, 23 March 2013, around Gangnani (2,200 m asl) in oak-rhododendron forest, also feeding on grasses along roads. They were sighted in small groups of 4-5 individuals in winter around Uttarkashi (1,300 m asl) feeding from leaf buds and berries and seeds of *Urtcica dioica*.

Black and Yellow Grosbeak (*Mycerobas icterioides*): Sighted usually in pairs around Harsil at an elevation of 2,700 m asl in summer, 2014 and 2015 in moss covered boughs of *Cedrus deodara*, feeding on shrubs and collecting nest material. A pair was also spotted around Bhatwadi in a patch of *Alnus nepalenis* on multiple days in February 2017.

Spot-winged Grosbeak (*Mycerobas melanoxanthos*) (Fig. 5d): Seen in huge flocks (10-13 individuals, male-dominated in numbers) around Maneri (1,400 m asl) in winter and spring months. Very vocal during feeding and flying, the flock feeds on one tree at a time .

Crested Bunting (*Melophus lathami*): A pair of birds were seen foraging along charred grassy patches near a perennial stream (joining the main river) along a stony path used by villagers, in Devprayag. They were often resting on pebbles, rocks and bushes or thorn thickets. Their body colour was concealed by the surroundings.

Red-fronted Serin (Fig. 5c): A group of seven birds were seen around village areas in Harsil (2,700 m asl), foraging from fruiting trees and thistles in March 2014. A smaller flock was regularly sighted during winters around Uttarkashi area (1,300-1,500 m asl) feeding on berries in shrubs.

Himalayan Beautiful Rosefinch (Fig. 4f): A single bird was seen on a snowy day in February 2014 at an elevation of 2,600 m asl (around Dharali) in an orchard by the river bank. The bird made frequent calls with frequent short sallying movements in air. A flock of 4-5 individuals was seen again in the same vicinity on 16 March 2018.

## Discussion

Riverine habitats are important for birds globally, with around 60 specialist species recognised and up to 23% of all bird species utilising freshwaters, including rivers, for part or all of their life cycles (Buckton 1998, Buckton and Ormerod 2002, Ormerod and Tyler 1993). The present study reveals that riverine areas along Himalayan headwaters hold a rich avian community with a representation from 64 families. This corroborates that riverine areas provide a range of habitats required for species belonging to different families. Qualitative field studies like ours can potentially provide the baseline data for ecological questions pertaining to the effects of habitat modification apart from understanding the basic ecology of individual species or communities. Natural habitats are undergoing rapid modification owing to multiple stressors and documenting information on wildlife residing in natural versus modified habitats can provide insight to management needs. Recognition of the riparian corridor as significant areas of maintaining regional biodiversity holds promise for issues related to watershed management. Alteration of river flow regimes is a global concern in terms of maintaining the integrity of these land-water ecotone habitats (Naiman et al. 1993). Forests along headwater streams may be important habitats for many species. Unfortunately, in India, the location of most dams overlap with species-rich areas in the Himalaya (Pandit and Grumbine 2012).

These habitats are crucial for riverine specialists. We documented seven riverine obligate species: White-capped Redstart (*Phoenicurus leucocephalus*), Plumbeous water Redstart (*Phoenicurus fuliginosus*), Little Forktail (*Enicurus scouleri*), Spotted Forktail (*Enicurus maculatus*), Brown Dipper (*Cinclus pallasi*), Crested Kingfisher (*Megaceryle lugubris*) and Ibisbill (*Ibidoryncha struthersii*). Many others used the riverine habitats opportunistically. The Grey Wagtail (*Motacilla cinerea*), Common Sandpiper (*Actitis hyoleucos*) and White Wagtail (*Motacilla alba*) were found to be breeding on higher elevation river banks. Birds like the Common Kingfisher (*Alcedo atthis*), White-throated Kingfisher (*Halcyon smyrenensis*) and River Lapwing (*Vanellus duvaucelli*) feed substantially from river production although they are found along inland waters as well. The studied bird community constituted a large number of terrestrial species (n=227) as well as water-dependent species (n=51). The bird community shows a predominance of species (n=30) from the Muscicapidae family probably owing to the fact that riparian areas produce higher numbers of insects (Gray 1993, Jackson and Fisher 1986) than surrounding habitats. In the present study, a good number (n=11) of IUCN Red-listed species were recorded (Table 2) (Praveen et al. 2017).

Understanding species habitat requirements is imperative in guiding management recommendations for conservation planning, as it may help to reduce the division often apparent between modellers and conservation practitioners. Observational field studies, like ours, lay the foundation for the same by documentaing species distribution for areas which lie outside protected areas. Shifts in the structure and function of many freshwater ecosystems are attributed to climatic changes leading to decreases in primary productivity and uncoupling of trophic linkages along with shifts in the composition of riverine communities. This renders these riverine ecosystems and dependent flora and fauna vulnerable to ecological malfunctioning and ultimately biodiversity loss. Specifically in our study area, due to the development of the Tehri dam and Koteshwar hydropower plant, around 153 km (almost 71%) of river length has been affected. Bank-nesting species are vulnerable to loss of riparian habitat and nest flooding during sensitive periods of their annual cycles such as breeding (Chiu et al. 2008, [Bibr B4736892][Bibr B4776004]). Riverine areas not only provide remnant habitats for many habitat specialists discussed above, but also provide corridors between otherwise isolated pockets of habitats. Conflicts between biodiversity conservation and ecosystem services provided by riverine areas may ultimately arise, with global freshwater resources likely to be further stressed due to increasing demand for water needed to sustain growing human populations and changing climate. As many riverine forest sites are bound to undergo irreversible changes, conservation efforts focused at a large spatial scale with considerations for natural fluvial geomorphic processes should be prioritised.

## Supplementary Material

Supplementary material 1 Number of species from each family Data type: Table and graph
Brief description: This table and the bar graph summarises the number of bird species from each family
File: oo_265596.xlsx Ankita Sinha and Hima Hariharan

## Figures and Tables

**Figure 4a.**
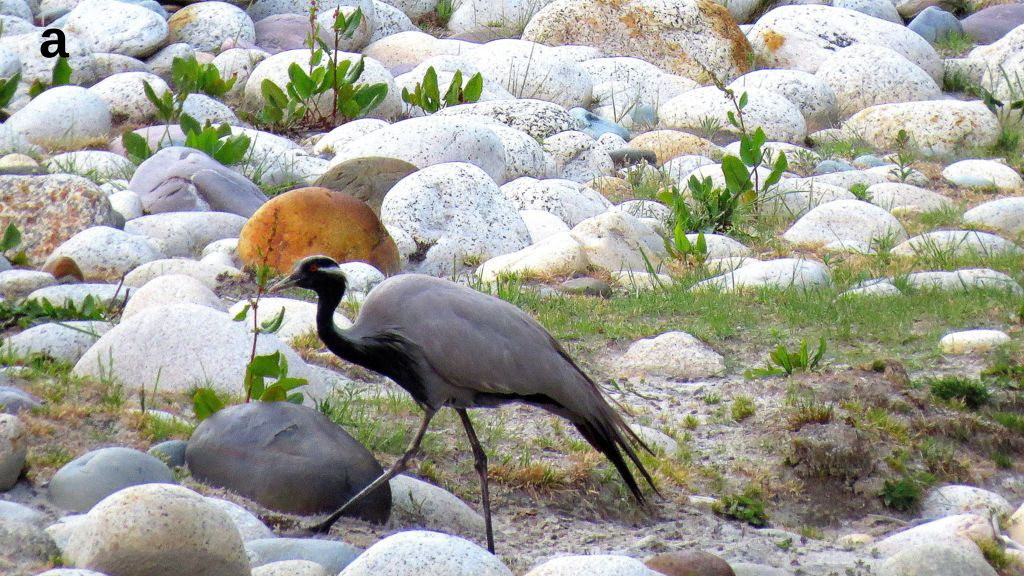
Demoiselle Crane (Photo by Ankita Sinha)

**Figure 4b.**
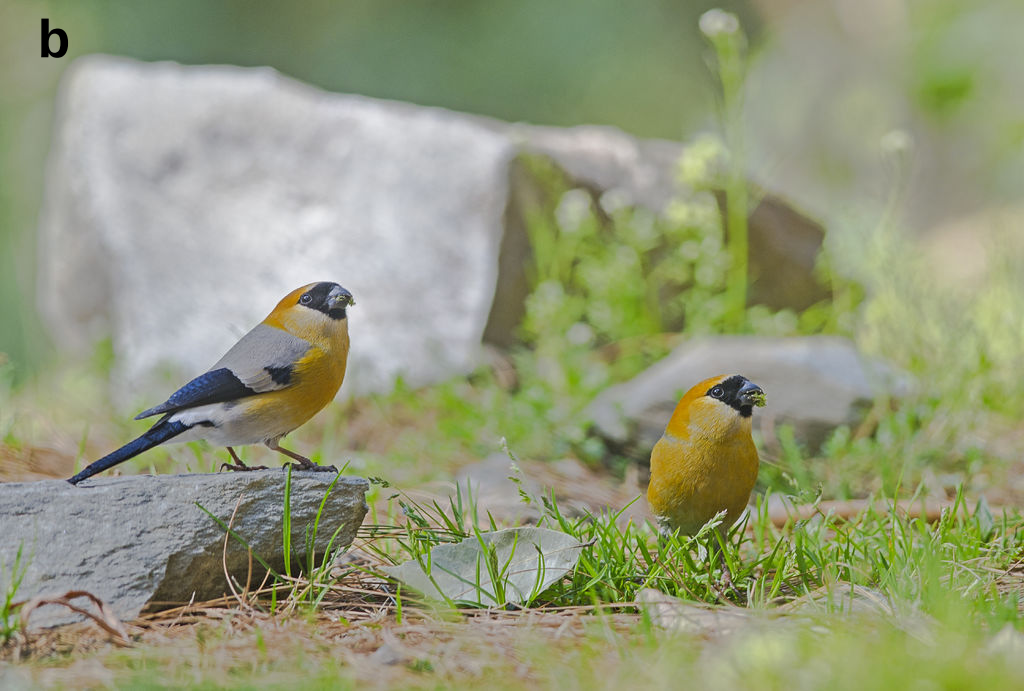
Red-headed Bullfinch (Photo by Nilanjan Chatterjee)

**Figure 4c.**
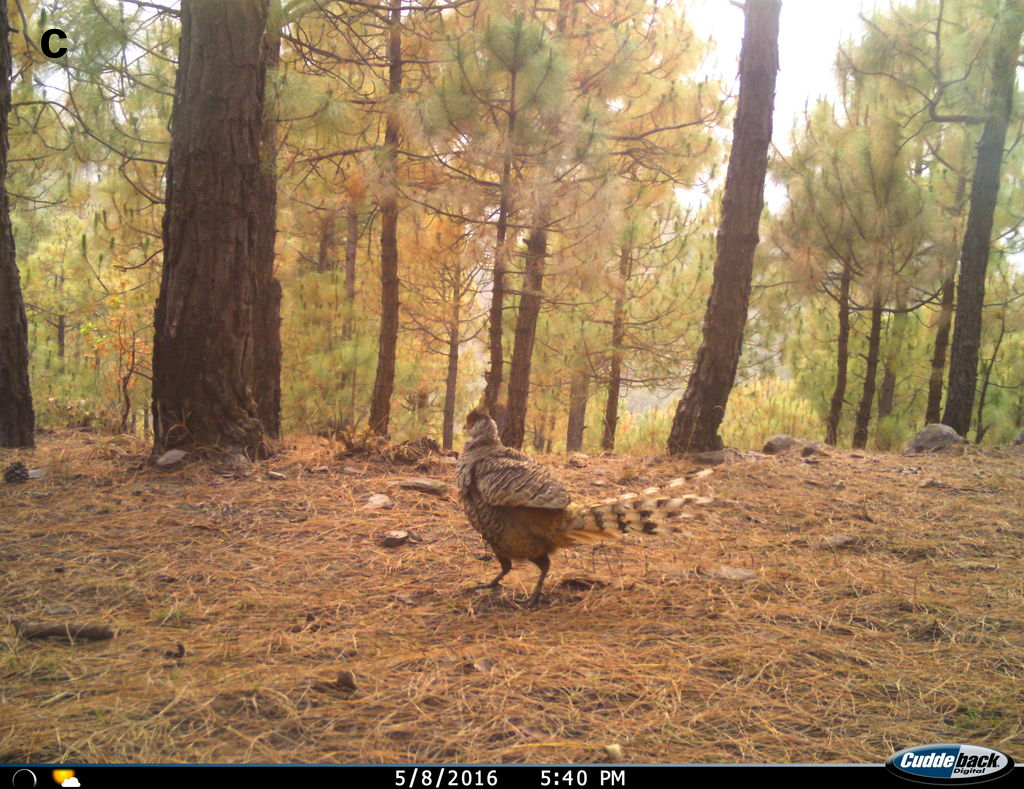
Cheer Pheasant (Camera trap photograph shared by Meghna Bandopadhyay)

**Figure 4d.**
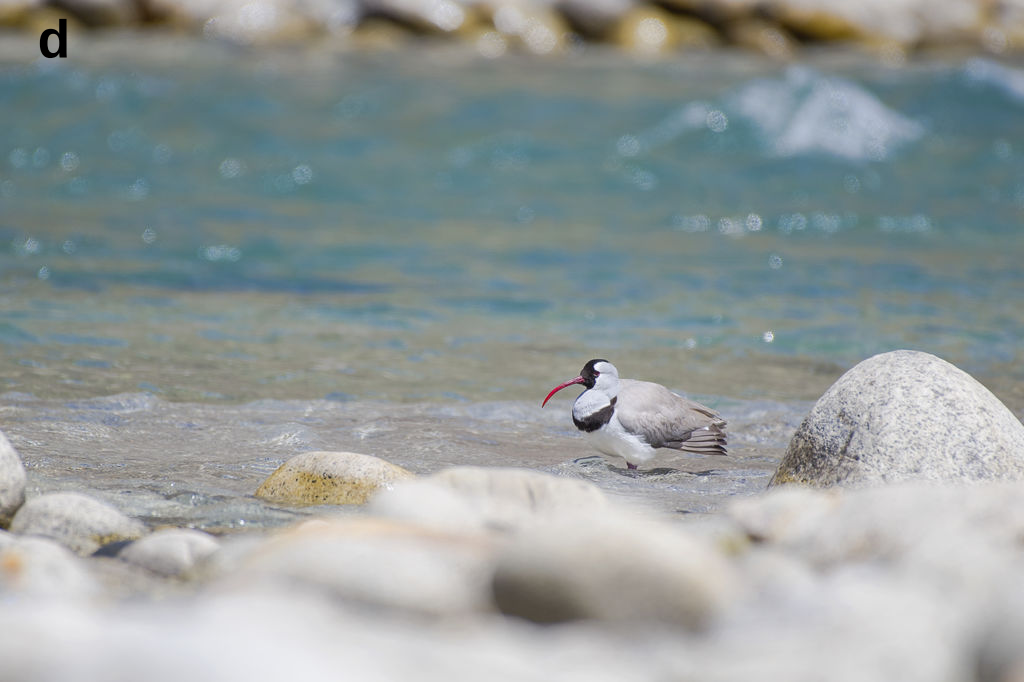
Ibisbill (Photo Nilanjan Chatterjee)

**Figure 4e.**
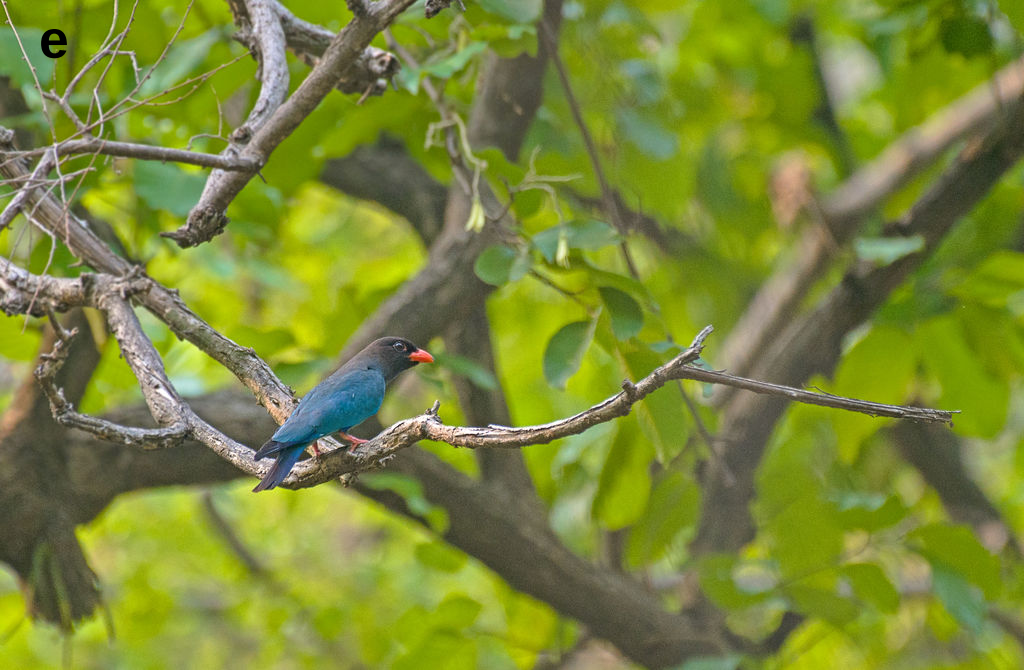
Dollarbird (Photo by Nilanjan Chatterjee)

**Figure 4f.**
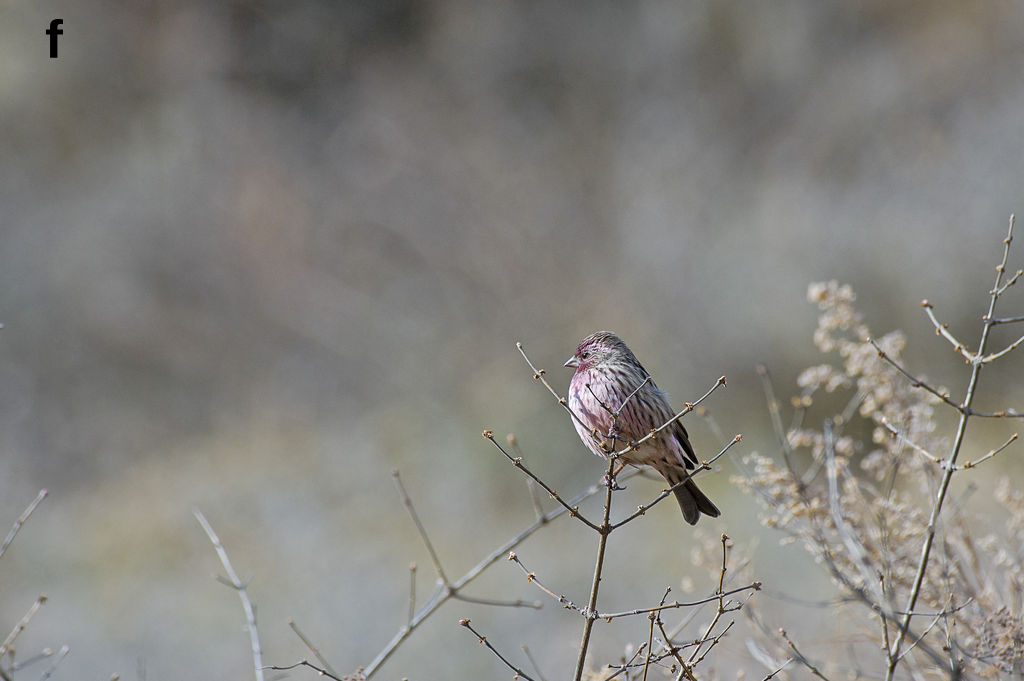
Himalayan Beautiful Rosefinch (Photo by Nilanjan Chatterjee)

**Figure 5a.**
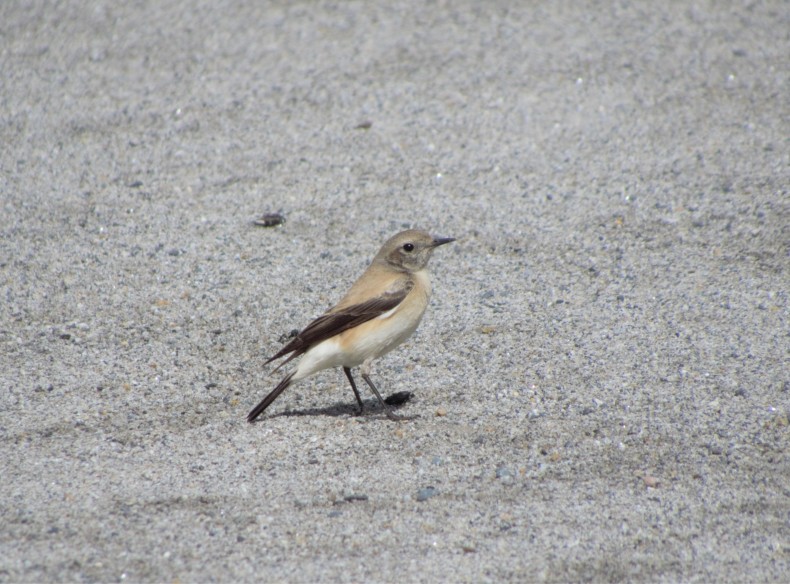
Desert Wheatear (Photo Ankita Sinha)

**Figure 5b.**
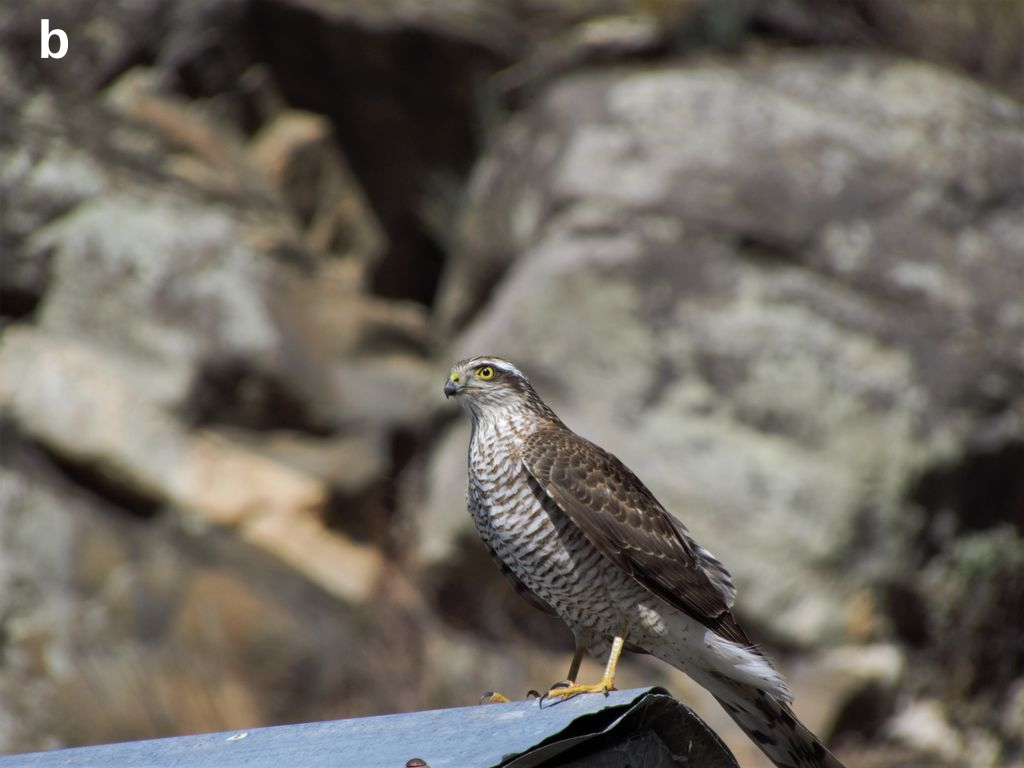
Northern Goshawk (Photo Ankita Sinha)

**Figure 5c.**
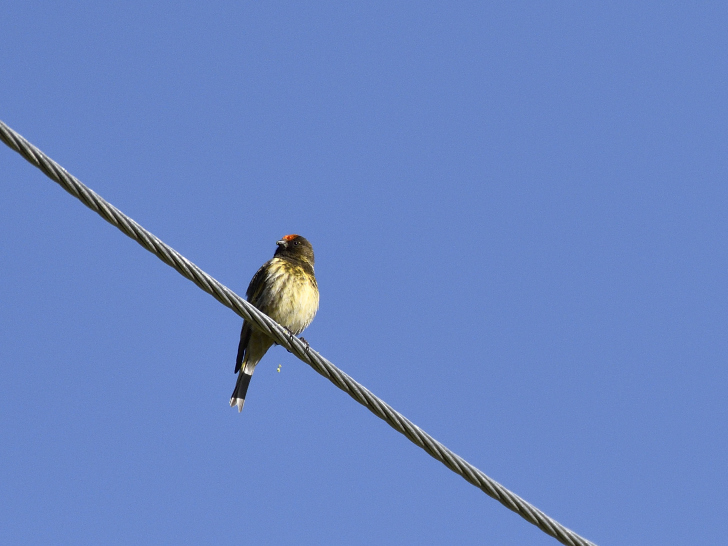
Red-fronted Serin (Photo by Nilanjan Chatterjee)

**Figure 5d.**
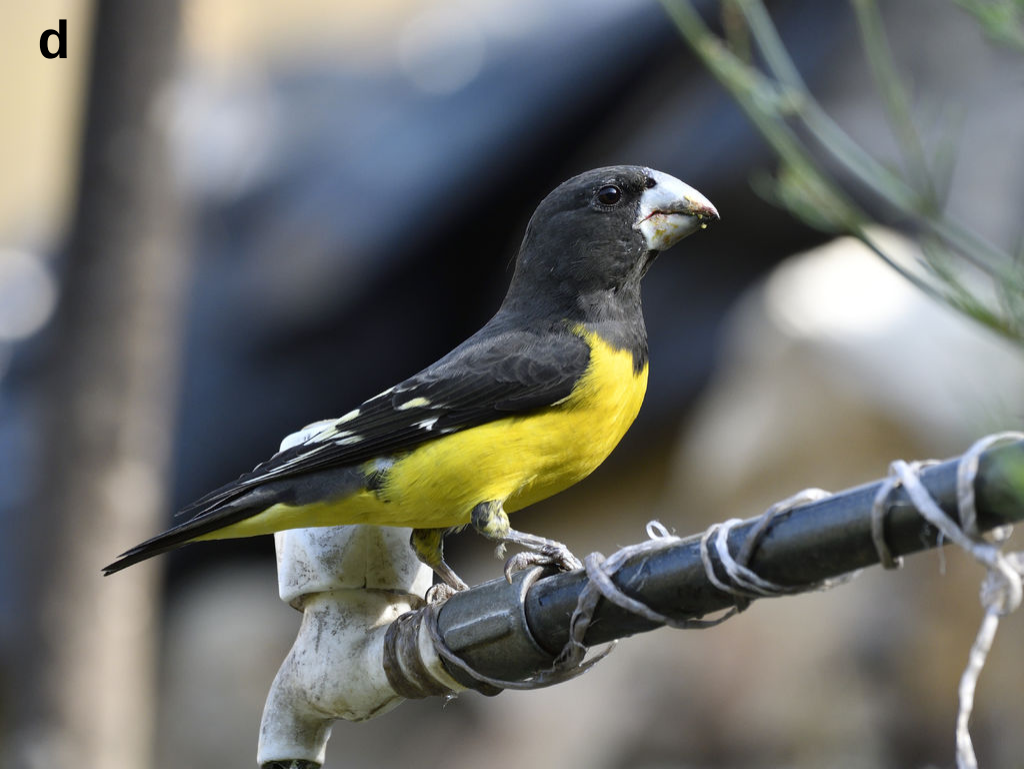
Spot-winged Grosbeak (Photo by Nilanjan Chatterjee)

**Figure 5e.**
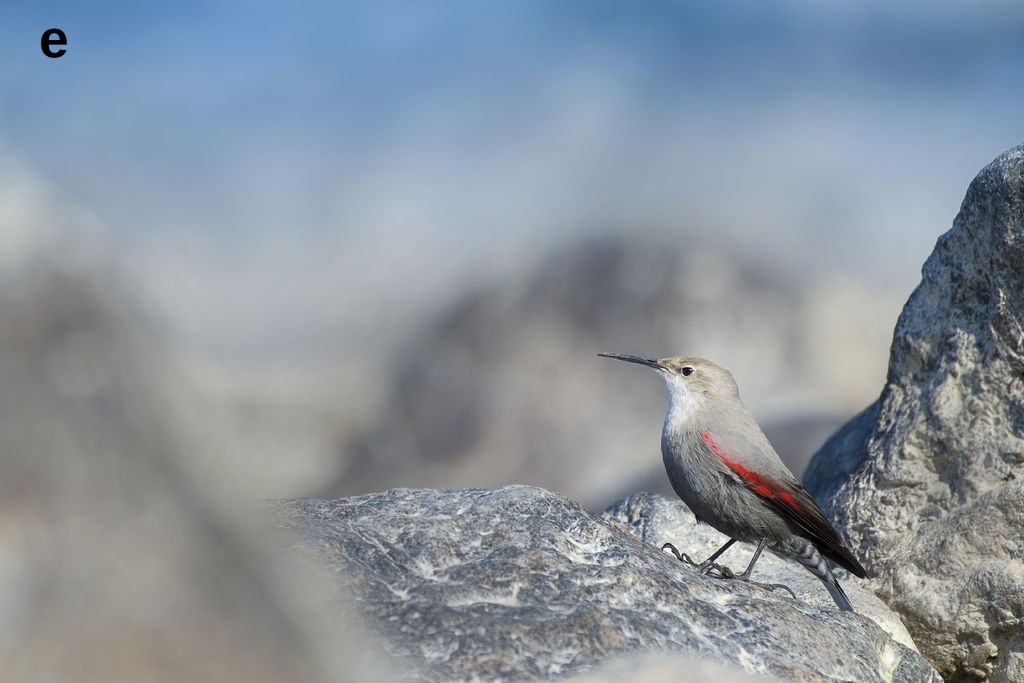
Wallcreeper (Photo by Nilanjan Chatterjee)

**Figure 5f.**
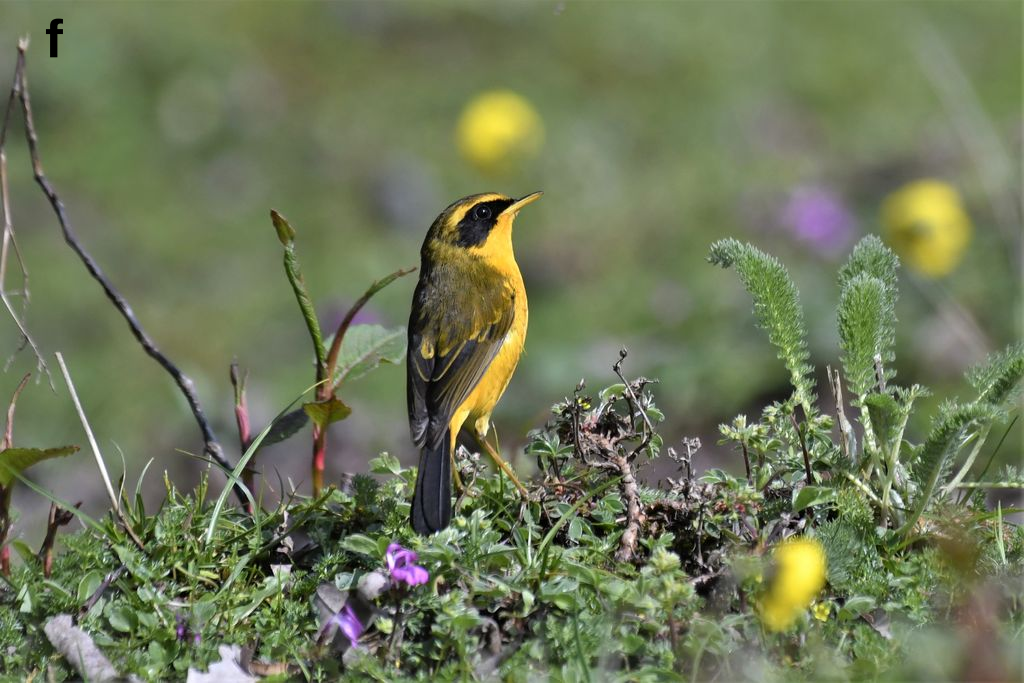
Golden Bush Robin (Photo by Nilanjan Chatterjee)

**Table 1.** List of species recorded during the study period. Elevational distribution (in parentheses and measured in metres) for species regularly seen in the study area: * All Seasons, # Summer, √ Winter, α Passage migrant and β Vagrant following Praveen et al. (2018)

**Common name**	**Scientific name**	**Rishikesh** **(300)**	**Devprayag (700)**	**Tehri** **(2100)**	**Uttarkashi (1300)**	**Harsil (2500)**	**Gangotri** **(3200)**
Ruddy Shelduck	*Tadorna ferruginea* (Pallas, 1764)	√		√	√	√	
Red-crested Pochard	*Netta rufina* (Pallas, 1773)				√		
Common Pochard	*Aythya farina* (Linnaeus, 1758)			√	√		
Ferruginous Duck	*Aythya nyroca* (Güldenstädt, 1770)				√		
Northern Shoveller	*Spatula clypeata* (Linnaeus, 1758)				√	√	
Gadwall	*Mareca strepera* (Linnaeus, 1758)				√		
Eurasian Wigeon	*Mareca penelope* (Linnaeus, 1758)				√		
Indian Spot-billed Duck	*Anas poecilorhyncha* (J.R. Forster, 1781)				√		
Mallard	*Anas platyrynchos* (Linnaeus, 1758)				√		
Northern Pintail	*Anas acuta* (Linnaeus, 1758)				√		
Common Teal	*Anas crecca* (Linnaeus, 1758)				√		
Common Hill Patridge	*Arborophila torqueola* (Valenciennes, 1825)				#	#	
Indian Peafowl	*Pavo cristatus* (Linnaeus, 1758)	*					
Chukar Patridge	*Alectoris chukar* (J.E. Gray, 1830)				√	#	#
Snow Patridge	*Lerwa lerwa* (Hodgson, 1833)						#
Black Francolin	*Francolinus francolinus* (Linnaeus, 1766)		√	*	*		
Red Junglefowl	*Gallus gallus* (Linnaeus, 1758)	*	*	*	*		
Himalayan Monal	*Lophophorus impejanus* (Latham, 1790)					√	
Cheer Pheasant	*Catreus wallichi* (Hardwicke, 1827)		#		√		
Kalij Pheasant	*Lophura leucomelanos* (Latham, 1790)	√	*	*	*		
Koklass Pheasant	*Pucrasia macrolopha* (Lesson, 1829)					√	
Common Pigeon	*Columba livia* (J.F. Gmelin, 1789)	*	*	*	*	*	*
Snow Pigeon	*Columba leuconota* (Vigors, 1831)					√	√
Oriental Turtle Dove	*Streptopelia orientalis* (Latham, 1790)	√	√	*	*	*	#
Eurasian Collared Dove	*Streptopelia decaocto* (Frivaldszky, 1838)	*					
Spotted Dove	*Stireptopelia chinensis* (Scopoli, 1786)	*					
Yellow-legged Green Pigeon	*Treron phoenicopterus* (Latham, 1790)	*			*		
Wedge-tailed Green Pigeon	*Treron sphenurus* (Vigors, 1832)				*		
Asian Emerald Dove	*Chalcophaps indica* (Linnaeus, 1758)	*					
Large-tailed Nightjar	*Caprimulgus macrurus* (Horsfield, 1821)	#	#				
Indian Nightjar	*Caprimulgus asiaticus* (Latham, 1790)				#		
Himalayan Swiftlet	*Aerodramus brevirostris* (Horsfield, 1840)				*		
Indian House Swift	*Apus affinis* (J.E. Gray, 1830)			*	*		
Greater Coucal	*Centropus sinensis* (Stephens, 1815)	*					
Asian Koel	*Eudynamys scolopaceus* (Linnaeus, 1758)	*	#		#		
Large Hawk Cuckoo	*Hierococcyx sparverioides* (Vigors, 1832)	#	#	#	#	#	
Common Hawk Cuckoo	*Hierococcyx varius* (Vahl, 1797)	*					
Common Cuckoo	*Cuculus canorus* (Linnaeus, 1758)					#	
Himalayan Cuckoo	*Cuculus saturates* (Blyth, 1843)				#	#	
Demoiselle Crane	*Grus virgo* (Linnaeus, 1758)					β	
Black-crowned Night Heron	*Nycticorax nycticorax* (Linnaeus, 1758)	*					
Striated Heron	*Butorides striata* (Linnaeus, 1758)	#					
Indian Pond Heron	*Ardeola grayii* (Sykes, 1832)					√	
Cattle Egret	*Bubulcus ibis* (Linnaeus, 1758)	*					
Grey Heron	*Ardea cinerea* (Linnaeus, 1758)	√					
Great Egret	*Ardea alba* (Linnaeus, 1758)	*					
Intermediate Egret	*Ardea intermedia* (Wagler, 1829)	*					
Little Egret	*Egretta garzetta* (Linnaeus, 1766)	*					
Little Cormorant	*Microcarbo niger* (Vieillot, 1817)	*	*				
Great Cormorant	*Phalacrocorax carbo* (Linnaeus, 1758)	α	α	α	α	α	
Great Thick-knee	*Esacus recurvirostris* (Cuvier, 1829)	#					
Ibisbill	*Ibidoryncha struthersii* (Vigors, 1832)					*	
River Lapwing	*Vanellus duvaucelli* (Lesson, 1826)	*	*				
Red-wattled Lapwing	*Vanellus indicus* (Boddaert, 1783)	*	*	*	*		
Common Sandpiper	*Actitis hypoleucos* (Linnaeus, 1758)		√		√	#	#
Green Sandpiper	*Tringa ochropus* (Linnaeus, 1758)				√		
Pallas's Gull	*Ichthyaetus ichthyaetus* (Pallas, 1773)	√					
Osprey	*Pandion haliaetus* (Linnaeus, 1758)				√		
Bearded Vulture	*Gypaetus barbatus* (Linnaeus, 1758)			√	√	*	*
Egyptian Vulture	*Neophron percnopterus* (Linnaeus, 1758)			*	*		
Crested Serpent Eagle	*Spilornis cheela* (Latham, 1790)	*					
Himalayan Vulture	*Gyps himalayensis* (Hume, 1869)				√	*	*
Griffon Vulture	*Gyps fulvus* (Hablizl, 1783)		√	√	√	*	*
Mountain Hawk Eagle	*Nisaetus nipalensis* (Hodgson, 1836)				#		
Changeable Hawk Eagle	*Nisaetus cirrhatus* (J.F. Gmelin, 1788)	*					
Steppe Eagle	*Aquila nipalensis* (Hodgson, 1833)				*	*	
Golden Eagle	*Aquila chrysaetos* (Linnaeus, 1758)						*
Hen Harrier	*Circus cyaneus* (Linnaeus, 1766)					√	
Shikra	*Accipiter badius* (J.F. Gmelin, 1788)	*	*				
Eurasian Sparrowhawk	*Accipiter nisus* (Linnaeus, 1758)						*
Northern Goshawk	*Accipiter genitilis* (Linnaeus, 1758)					√	
Pallas's Fish Eagle	*Haliaeetus leucoryphus* (Pallas, 1771)				*		
Black Kite	*Milvus migrans* (Boddaert, 1783)	*	*	*	*		
Black-eared Kite	*Milvus migrans lineatus* (Boddaert, 1783)	*	*	*	*	√	
White-eyed Buzzard	*Butastur teesa* (Franklin, 1831)				√		
Himalayan Buzzard	*Buteo refectus* (Portenko, 1935)				√	#	
Collared Owlet	*Glaucidium brodiei* (E. Burton, 1836)				*		
Asian Barred Owlet	*Glaucidium cuculoides* (Vigors, 1831)		*	#	#		
Spotted Owlet	*Athene brama* (Temminck, 1821)						
Brown Fish Owl	*Ketupa zeylonensis* (J.F. Gmelin, 1788)	*					
Indian Grey Hornbill	*Ocyceros birostris* (Scopoli, 1786)	*					
Common Hoopoe	*Upupa epops* (Linnaeus, 1758)	*	*	#	*	#	
Speckled Piculet	*Picumnus innominatus* (E. Burton, 1836)		√	*	*		
Himalayan Golden-backed Woodpecker	*Dinopium shorii* (Vigors, 1831)	*					
Lesser Golden-backed Woodpecker	*Dinopium benghalense* (Linnaeus, 1758)	*					
Greater Yellownape	*Chrysophlegma flavinucha* (Gould, 1834)		*	*			
Lesser Yellow-naped Woodpecker	*Picus chlorolophus* (Vieillot, 1818)		*	*	#		
Grey-headed Woodpecker	*Picus canus* (J.F. Gmelin, 1788)		*	*	*	#	
Scaly-bellied Woodpecker	*Picus squamatus* (Vigors, 1831)		*	*	*	*	
Grey-capped Pygmy Woodpecker	*Dendrocopos canicapillus* (Blyth, 1845)		√	*	*		
Fulvuos-breasted Woodpecker	*Dendrocopos macei* (Vieillot, 1818)		*		#		
Brown-fronted Woodpecker	*Dendrocopos auriceps* (Vigors, 1831)	*					
Yellow-crowned Woodpecker	*Dendrocopos mahrattensis* (Latham, 1801)	#					
Himalayan Woodpecker	*Dendrocopos himalayensis* (Jardine & Selby, 1831)			*	#		
Rufous-bellied Woodpecker	*Dendrocopos hyperythrus* (Vigors, 1831)				#		
Great Barbet	*Psilopogon virens* (Boddaert, 1783)	√	*	*	*	#	
Brown-headed Barbet	*Psilopogon zeylanicus* (J.F. Gmelin, 1788)	*					
Lineated Barbet	*Psilopogon lineatus* (Vieillot, 1816)	*					
Blue-throated Barbet	*Psilopogon asiaticus* (Latham, 1790)			*	*		
Green Bee-eater	*Merops orientalis* (Latham, 1801)	*	*	*			
Chestnut-headed Bee-eater	*Merops leschenaulti* (Vieillot, 1817)			*	*		
Dollarbird	*Eurystomus orientalis* (Linnaeus, 1766)		*				
Common Kingfisher	*Alcedo atthis* (Linnaeus, 1758)	*	*		*		
Crested Kingfisher	*Megaceryle lugubris* (Temminck, 1834)	*	*	*	*		
Pied Kingfisher	*Ceryle rudis* (Linnaeus, 1758)	*					
White-throated Kingfisher	*Halcyon smyrnensis* (Linnaeus, 1758)	*	*	*	*		
Common Kestrel	*Falco tinnunculus* (Linnaeus, 1758)	*	*	*	*	*	
Eurasian Hobby	*Falco subbuteo* (Linnaeus, 1758)						√
Peregrine Falcon	*Falco peregrinus* (Tunstall, 1771)			*	*		
Slaty-headed Parakeet	*Psittacula himalayana* (Lesson, 1832)	√	√		*		
Plum-headed Parakeet	*Psittacula cyanocephala* (Linnaeus, 1766)	√	√	*	*		
Alexandrine Parakeet	*Psittacula eupatria* (Linnaeus, 1766)	*					
Rose-ringed Parakeet	*Psittacula krameri* (Scopoli, 1769)	*	*	*	*		
Long-tailed Minivet	*Pericrocotus ethologus* (Bangs & J.C. Phillips, 1914)	√	√		*	#	
Scarlet Minivet	Pericrocotus (flammeus) speciosus (J.R. Forster, 1781)	√	√		*		
Large Cuckooshrike	*Coracina javensis* (Horsfield, 1821)	*					
Himalayan Shrike-babbler	*Pteruthius ripleyi* (Biswas, 1960)				√		
Green Shrike-babbler	*Pteruthius xanthochlorus* (J.E. & G.R. Gray, 1847)				√		
Maroon Oriole	*Oriolus trailii* (Vigors, 1832)	√	√		*		
Black-hooded Oriole	*Oriolus xanthornus* (Linnaeus, 1758)	*					
Indian Golden Oriole	Oriolus (oriolus) kundoo (Sykes, 1832)				#		
Bar-winged Flycatcher-Shrike	*Hemipus picatus* (Sykes, 1832)	√					
Black Drongo	*Dicrurus macrocercus* (Vieillot, 1817)	*					
Ashy Drongo	*Dicrurus leucophaeus* (Vieillot, 1817)		√	#	#	#	
Hair-crested Drongo	*Dicrurus hottentottus* (Linnaeus, 1766)	*	*		*		
White-throated Fantail	*Rhipidura albicollis* (Vieillot, 1818)	√	√		#		
Bay-backed Shrike	*Lanius vittatus* (Valenciennes, 1826)	*					
Long-tailed Shrike	*Lanius schach* (Linnaeus, 1758)	*	*	*	*	*	
Grey-backed Shrike	*Lanius tephronotus* (Vigors, 1831)	√					
Rufous Treepie	*Dendrocitta vagabunda* (Latham, 1790)	*	*				
Grey Treepie	*Dendrocitta formosae* (Swinhoe, 1863)	√	*	*	*	*	
Red-billed Chough	*Pyrrhocorax pyrrhocorax* (Linnaeus, 1758)						#
Yellow-billed Chough	*Pyrrhocorax graculus* (Linnaeus, 1766)						*
Yellow-billed Blue Magpie	*Urocissa flavirostris* (Blyth, 1846)	*	*	#	#		
Red-billed Blue Magpie	*Urocissa erythrorhyncha* (Boddaert, 1783)	√	*	*	*	#	
Eurasian Jay	*Garrulus glandarius* (Linnaeus, 1758)				*		
Black-headed Jay	*Garrulus lanceolatus* (Vigors, 1830)				*		
Spotted Nutcracker	*Nucifraga caryocatactes* (Linnaeus, 1758)					#	
House Crow	*Corvus splendens* (Vieillot, 1817)	*	*	*	*		
Large-billed Crow	*Corvus macrorhynchos* (Wagler, 1827)				*	*	*
Indian Paradise-flycatcher	*Terpsiphone paradisi* (Linnaeus, 1758)	#	#	#	#		
Pale-billed Flowerpecker	*Dicaeum erythrorynchos* (Latham, 1790)	*	#				
Fire-breasted Flowerpecker	*Dicaeum ignipectus* (Latham, 1790)		√	*	*		
Purple Sunbird	*Cinnyris asiaticus* (Latham, 1790)	*	*	*	#		
Black-throated Sunbird	*Aethopyga saturate* (Hodgson, 1836)				β		
Crimson Sunbird	*Aethopyga siparaja* (Raffles, 1822)	*	*	*	#		
Golden-fronted Leafbird	*Chloropsis aurifrons* (Temminck, 1829)		#				
Rufous-breasted Accentor	*Prunella strophiata* (Blyth, 1843)				√	#	
Blak-throated Accentor	*Prunella atrogularis* (von Brandt, 1843)				√	*	
White-rumped Munia	*Lonchura striata* (Linnaeus, 1766)		*	*	*		
Scaly-breasted Munia	*Lonchura punctulata* (Linnaeus, 1758)	*	*	#	#		
House Sparrow	*Passer domesticus* (Linnaeus, 1758)	*	*	*	*	*	*
Russet Sparrow	*Passer cinnamomeus* (Gould, 1836)	√	√	*	*	#	#
Yellow-throated Sparrow	*Gymnoris xanthocollis* (E. Burton, 1838)	#	#				
Olive-backed Pipit	*Anthus hodgsoni* (Richmond, 1907)					#	
Rosy Pipit	*Anthus roseatus* (Blyth, 1847)					#	
Paddyfield Pipit	*Anthus rufulus* (Vieillot, 1818)	#			#		
Grey Wagtail	*Motacilla cinerea* (Tunstall, 1771)	√	√	#	*	*	#
Citrine Wagtail	*Motacilla citreola* (Pallas, 1776)	#					
White-browed Wagtail	*Motacilla maderaspatensis* (J.F. Gmelin, 1789)	*	*	#	#		
White Wagtail	*Motacilla alba* (Linnaeus, 1758)					#	#
Black-and-yellow Grosbeak	*Mycerobas icterioides* (Vigors, 1831)				#	#	
Collared Grosbeak	*Mycerobas affnis* (Blyth, 1855)				√		
Spot-winged Grosbeak	*Mycerobas melanozanthos* (Hodgson, 1836)				*		
Common Rosefinch	*Carpodacus erythrinus* (Pallas, 1770)			√	√	#	
Himalayan Beautiful Rosefinch	*Carpodacus pulcherrimus* (F. Moore, 1856)					√	
Pink-browed Rosefinch	*Carpodacus rodochroa* (Vigors, 1831)			√	√	#	
Spot-winged Rosefinch	*Carpodacus rodopeplus* (Vigors, 1831)				#		
Red-headed Bullfinch	*Pyrrhula erythrocephala* (Vigors, 1832)				√	#	
Dark-breasted Rosefinch	*Procarduelis nipalensis* (Hodgson, 1836)			√	√	#	
Plain Mountain Finch	*Leucosticte nemoricola* (Hodgson, 1836)					√	
Yellow-breasted Greenfinch	*Chloris spinoides* (Vigors, 1831)	√	√	#	#		
European Goldfinch	*Carduelis carduelis* (Linnaeus, 1758)					#	
Fire-fronted Serin	*Serinus pusillus* (Pallas, 1811)				√	#	
Crested Bunting	*Melophus lathami* (J.E. Gray, 1831)		#				
Rock Bunting	*Emberiza cia* (Linnaeus, 1766)				√	#	#
Yellow-bellied Fairy Fantail	*Chelidorhynx hypoxantha* (Blyth, 1843)	√	√	*	*	#	#
Grey-headed Canary-flycatcher	*Culicicapa ceylonensis* (Swainson, 1820)	√	√	#	#	#	
Coal Tit	*Periparus ater(melanolophus)* (Linnaeus, 1758)					#	#
Rufous-naped Tit	*Periparus rufonuchalis* (Blyth, 1849)					#	#
Rufous-vented Tit	*Periparus rubidiventris* (Blyth, 1847)					#	#
Green-backed Tit	*Parus monticolus* (Vigors, 1831)	√	√	*	*	#	
Cinereous Tit	*Parus cinereus* (Vieillot, 1818)	√	√	#	*	#	
Black-lored Tit	*Machlolophus xanthogenys* (Vigors, 1831)				*	#	
Striated Prinia	*Prinia crinigera* (Hodgson, 1836)				*		
Grey-breasted Prinia	*Prinia hodgsonii* (Blyth, 1844)	*	*	*	*		
Ashy Prinia	*Prinia socialis* (Sykes, 1832)	*					
Plain Prinia	*Prinia inornata* (Sykes, 1832)				*		
Common Tailorbird	*Orthotomus sutorius* (Pennant, 1769)	*	*	*	*		
Scaly-breasted Wren Babbler	*Pnoepyga albiventer* (Hodgson, 1837)	*					
Streak-throated Swallow	*Petrochelidon fluvicola* (Blyth, 1855)	*					
Red-rumped Swallow	*Cecropis daurica* (Laxmann, 1769)			*	*		
Wire-tailed Swallow	*Hirundo smithii* (Leach, 1818)	#	#				
Barn Swallow	*Hirundo rustica* (Linnaeus, 1758)		#	#	#		
Dusky Crag Martin	*Ptyonoprogne concolor* (Sykes, 1832)	*					
Grey-throated Martin	*Riparia chinensis* (J.E. Gray, 1830)				*		
Black Bulbul	*Hipsypetes leucocephalus* (J.F. Gmelin, 1789)		√	*	*		
Red-whiskered Bulbul	*Pycnonotus jocosus* (Linnaeus, 1758)	√			#		
Himalayan Bulbul	*Pycnonotus leucogenis* (J.E. Gray, 1835)	*	*	*	*	#	
Red-vented Bulbul	*Pycnonotus cafer* (Linnaeus, 1766)	*	*	*	*		
Hume's Leaf Warbler	*Abrornis humei* (W.E. Brooks, 1878)	√	√	#	*	#	
Lemon-rumped Warbler	*Abrornis chloronotus* (J.E. & G.R. Gray, 1847)	√	√	#	#	#	
Buff-barred Warbler	*Abrornis pulcher* (Blyth, 1845)				#	#	
Tickell's Leaf Warbler	*Phylloscopus affinis* (Tickell, 1833)					#	#
Whistler's Warbler	*Seicercus whistleri* (Ticehurst, 1925)	√	√		#	√	
Greenish Warbler	*Seicercus trochiloides* (Sundevall, 1837)		α		Α	#	
Large-billed Leaf Warbler	*Seicercus magnirostris* (Blyth, 1843)					#	
Blyth's Leaf Warbler	*Seicercus reguloides* (Blyth, 1842)				#	#	
Western Crowned Leaf Warbler	*Seicercus occipitalis* (Blyth, 1845)					#	
Grey-hooded Leaf Warbler	*Seicercus xanthoschistos* (J.E. & G.R. Gray, 1847)	√	√	*	*	#	#
Grey-sided Bush Warbler	*Cettia brunnifrons* (Hodgson, 1845)				#		
Chestnut-headed Tesia	*Cettia castaneocoronata* (E. Burton, 1836)		√	#	#		
Black-faced Warbler	*Abroscopus schisticeps* (J.E. & G.R. Gray, 1847)			*			
Brown-flanked Bush Warbler	*Horornis forticeps* (Hodgson, 1845)				#		
Black-throated Tit	*Aegithalos concinnus* (Gould, 1855)		√	*	*	#	
Whiskered Yuhina	*Yuhina flavicollis* (Hodgson, 1836)	√	√	#	√	#	#
Oriental White-eye	Zosterops palpebrosus (Temminck, 1824)	*	*	*	*	#	
Rusty-cheeked Scimitar Babbler	*Erythrogenys erythrogenys* (Vigors, 1831)	*	*	*	*		
Black-chinned Babbler	*Cyanoderma pyrrhops* (Blyth, 1844)	*	*	*	*		
Puff-throated Babbler	*Pellorneum ruficeps* (Swainson, 1832)				*	*	
Striated Laughingthrush	*Grammatoptila striata* (Vigors, 1831)				*		
Jungle Babbler	*Turdoides striata* (Dumont, 1823)	*	*	*	*		
White-crested Laughingthrush	*Garrulax leucolophus* (Hardwicke, 1816)	*	*				
Rufous-chinned Laughingthrush	*Garrulax rufogularis* (Gould, 1835)		#				
White-throated Laughingthrush	*Garrulax albogularis* (Gould, 1836)		√	#	#		
Streaked Laughingthrush	*Trochalopteron lineatum* (Vigors, 1831)		√	*	*	#	
Variegated Laughingthrush	*Trochalopteron variegatum* (Vigors, 1831)				√	*	*
Chestnut-crowned Laughingthrush	*Trochalopteron erythrocephalum* (Vigors, 1832)				*		
Rufous Sibia	*Heterophasia capistrata* (Vigors, 1831)		√	#	*		
Red-billed Leiothrix	*Leiothrix lutea* (Scopoli, 1786)	*	*				
Bar-tailed treecreeper	*Certhia himalayana* (Vigors, 1832)				#	#	#
Chestnut-bellied Nuthatch	Sitta(castanea) cinnamoventris (Blyth, 1842)	√	√	*			
White-tailed Nuthatch	*Sitta himalayensis* (Jardine & Selby, 1835)					√	#
Velvet-fronted Nuthatch	*Sitta frontalis* (Swainson, 1820)	√					
Wallcreeper	*Tichodroma muraria* (Linnaeus, 1766)	√	√	*	*		
Eurasian Wren	*Troglodytes troglodytes* (Linnaeus, 1758)					√	√
Asain Pied Starling	*Gracupica contra* (Linnaeus, 1758)	*					
Chestnut-tailed Starling	*Sturnia malabarica* (J.F. Gmelin, 1789)				#		
Common Myna	*Acridotheres tristris* (Linnaeus, 1766)	*	*		#		
Brown Dipper	*Cinclus pallasii* (Temminck, 1820)	√	√	*	*	*	#
Indian Robin	*Saxicoloides fulicatus* (Linnaeus, 1766)	*					
Oriental Magpie Robin	*Copsychus saularis* (Linnaeus, 1758)	*	*	*	*		
Dark-sided Flycatcher	*Muscicapa sibirica* (J.F. Gmelin, 1789)					#	
Asian Brown Flycatcher	*Muscicapa dauurica* (Pallas, 1811)					#	
Tickell's Blue Flycatcher	*Cyornis tickelliae* (Blyth, 1843)	#					
Rufous-bellied Niltava	*Niltava sundara* (Hodgson, 1837)	√	√	#	#	#	
Small Niltava	*Niltava macgrigoriae* (E. Burton, 1836)	√			*		
Verditer Flycatcher	*Eumyias thalassinus* (Swainson, 1838)	√	√	*	#	#	
Hodgsons's Blue Robin	*Luscinia phaenicuroides* (J.E. & G.R. Gray, 1847)						√
Little Forktail	*Enicurus scouleri* (Vigors, 1832)	√	√		*	#	
Spotted Forktail	*Enicurus maculatus* (Vigors, 1831)	√	*		#	#	
Blue Whistling Thrush	*Myophnus caeruleus* (Scopoli, 1786)	√	√	*	*	*	*
Golden Bush Robin	*Tarsiger crysaeus* (Hodgson, 1845)				*		
Himalayan Bush Robin	*Tarsiger rufilatus* (Hodgson, 1845)			√	*	#	
Rusty-tailed Flycatcher	*Ficedula ruficauda* (Swainson, 1838)				#		
Rufous-gorgeted Flycatcher	*Ficedula strophiata* (Hodgson, 1837)	√	√	*	*	#	
Ultramarine Flycatcher	*Ficedula superciliaris* (Jerdon, 1840)	√	√	#	#		
Slaty-blue Flycatcher	*Ficedula tricolor* (Hodgson, 1845)	√	√				
Blue-fronted Redstart	*Phoenicurus frontalis* (Vigors, 1831)			*	*	*	
Blue-capped Redstart	*Phoenicurus coeruleocephala* (Vigors, 1831)			√	√	*	#
White-capped Water Redstart	*Phoenicurus leucocephalus* (Vigors, 1831)	√	√	*	*	*	#
Plumbeous Water Redstart	*Phoenicurus fuliginosus* (Vigors, 1831)	√	*	*	*	*	#
Blue-capped Rock Thrush	*Monticola cincloryncha* (Vigors, 1831)			*	*		
Chestnut-bellied Rock Thrush	*Monticola rufiventris* (Jardine & Selby, 1833)			*	*	#	
Blue Rock Thrush	*Monticola solitarius* (Linnaeus, 1758)	#					
Siberian Stonechat	*Saxicola maurus* (Pallas, 1773)		√	*	*		
Pied Bushchat	*Saxicola caprata* (Linnaeus, 1766)					#	#
Grey Bushchat	*Saxicola ferreus* (J.E. & G.R. Gray, 1847)					#	#
Desert Wheatear	*Oenanthe deserti* (Temminck, 1825)						α
Grandala	*Grandala coelicolor* (Hodgson, 1843)					√	
Long-tailed Thrush	*Zoothera dixoni* (Seebohm, 1881)			*	*		
Alpine Thrush	*Zoothera mollissima* (Blyth, 1842)				√		
Scaly Thrush	*Zoothera dauma* (Latham, 1790)				√		
Orange-headed Thrush	*Geokichla citrina* (Latham, 1790)	#					
Mistle Thrush	*Turdus viscivorus* (Linnaeus, 1758)					√	√
Grey-winged Blackbird	*Turdus boulboul* (Latham, 1790)				*		
Tickell's Thrush	*Turdus unicolor* (Tickell, 1833)				*		
White-collared Blackbird	*Turdus albocintus* (Royle, 1840)			#	#	#	
Chestnut Thrush	*Turdus rubrocanus* (J.E. & G.R. Gray, 1847)				√		
Black-throated Thrush	*Turdus artrogularis* (Jarocki, 1819)		√	#	#		

**Table 2.** List of IUCN red-listed species that were encountered during the survey period along the riverine areas of the Bhagirathi river.

**Common Name**	**Scientific name**	**IUCN category**
Egyptian Vulture	*Neophron percnopterus*	Endangered
Steppe Eagle	*Aquila nipalensis*	Endangered
Pallas's Fish-eagle	*Haliaeetus leucoryphus*	Endangered
Cheer Pheasant	* Catreuswallichii *	Vulnerable
Common Pochard	*Aythya farina*	Vulnerable
Ferruginous Duck	*Aythya nyroca*	Near Threatened
Great Thick-knee	*Esacus recurvirostris*	Near Threatened
River Lapwing	*Vanellus duvaucelii*	Near Threatened
Himalayan Griffon	*Gyps himalayensis*	Near Threatened
Alexandrine Parakeet	*Psittacula eupatria*	Near Threatened
Bearded Vulture	*Gypaetus barbatus*	Near Threatened
